# Vitamin D Deficiency in Hip Fracture Patients: Outcome-Specific Associations and Post-Fracture Management—A Narrative Review

**DOI:** 10.3390/medicina62091794

**Published:** 2026-09-17

**Authors:** Hannah Lee, Jaehee Lee, Yejun Lee, Suk-Kyoon Song

**Affiliations:** 1 School of Medicine, Daegu Catholic University, Daegu 42472, Republic of Korea; leehn@cu.ac.kr (H.L.); congderi04@cu.ac.kr (J.L.); iyejun021@cu.ac.kr (Y.L.); 2 Department of Orthopaedic Surgery, School of Medicine, Daegu Catholic University Medical Center, Daegu 42472, Republic of Korea

**Keywords:** hip fractures, vitamin D deficiency, vitamin D supplementation, postoperative outcomes

## Abstract

*Background and Objectives:* Vitamin D deficiency is common in older adults with hip fracture and has been associated with adverse postoperative outcomes. However, heterogeneity in deficiency thresholds, outcome definitions, and patient populations has complicated the interpretation and clinical application of existing evidence. This narrative review aimed to summarize current evidence regarding perioperative vitamin D status, its associations with postoperative complications, mortality, functional recovery, and secondary fracture, and the role of vitamin D supplementation after hip fracture. *Materials and Methods:* Relevant studies were identified through PubMed searches and manual screening of reference lists. Cohort studies, randomized controlled trials, systematic reviews, meta-analyses, and clinical guidelines addressing vitamin D status, supplementation, hip fracture outcomes, fracture prevention, or postoperative recovery in older adults were narratively synthesized. *Results:* Low serum 25-hydroxyvitamin D was associated with selected early medical complications, mortality, impaired functional recovery, and secondary hip fracture, although these associations varied across outcomes. The strongest prognostic associations with mobility were observed in patients with severe deficiency (approximately ≤10–12 ng/mL), whereas mild insufficiency showed less consistent associations. Heterogeneity among studies reflected differences in deficiency thresholds, sampling timing, outcome definitions, follow-up duration, study populations, and adjustment for frailty and comorbidity. Vitamin D supplementation consistently increased serum 25-hydroxyvitamin D concentrations but did not consistently improve gait, postoperative complications, mortality, or recurrent fracture. Favorable outcomes reported with exercise- and nutrition-based multimodal interventions could not be attributed to vitamin D supplementation alone. *Conclusions:* Vitamin D deficiency should be interpreted within the broader context of frailty, nutritional status, muscle function, and skeletal health after hip fracture. Vitamin D assessment and correction should be integrated into comprehensive post-fracture care alongside osteoporosis treatment, adequate calcium and protein intake, rehabilitation, and fall prevention. However, clinical benefits of supplementation beyond correction of deficiency remain uncertain, and future studies should focus on patients with severe deficiency and clinically meaningful, patient-centered outcomes.

## 1. Introduction

**Figure 1:**
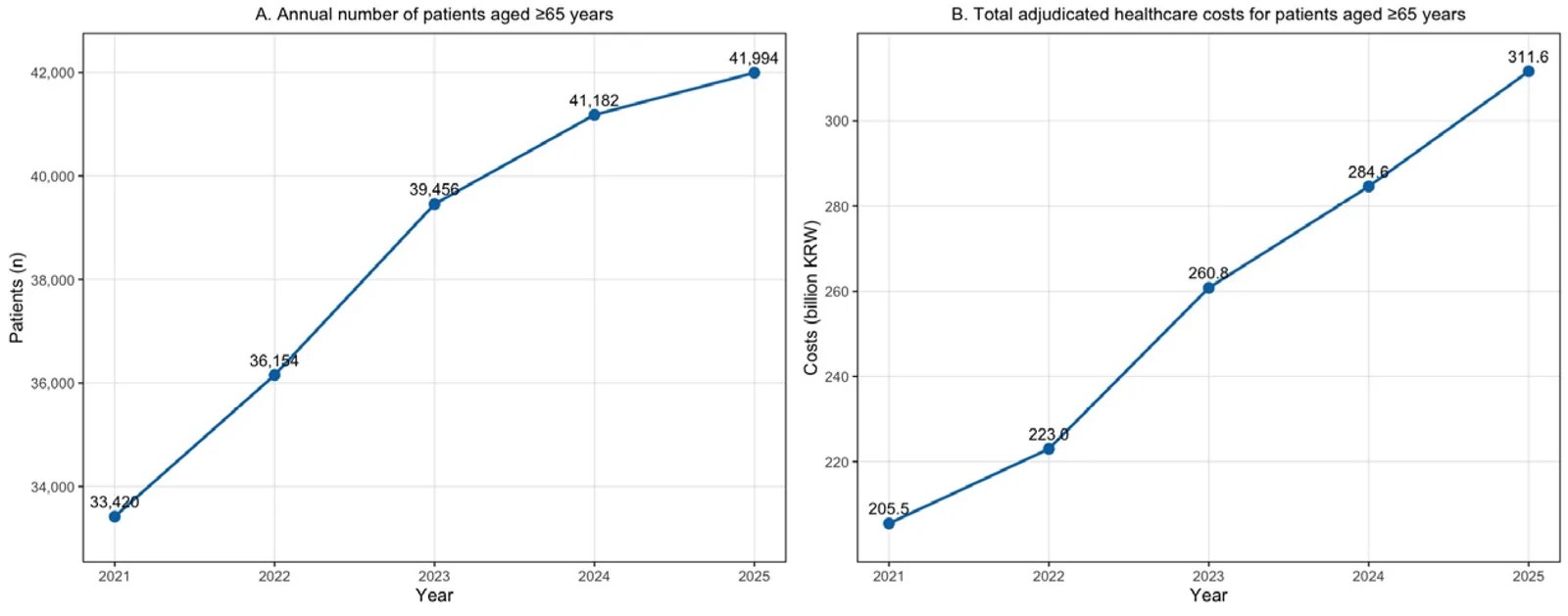
Trends in the claims-based burden of HIRA-defined hip fracture among adults aged 65 years or older in South Korea, 2021–2025.

Hip fracture is one of the most devastating osteoporotic fractures in older adults because it is associated with substantial postoperative morbidity, functional decline, loss of independence, institutionalization, and excess mortality. As populations age, the incidence and socioeconomic burden of hip fracture continue to increase worldwide. In South Korea, National Health Insurance claims data similarly demonstrate a steadily increasing burden of hip fracture among older adults. According to the Health Insurance Review and Assessment Service (HIRA), the annual number of patients aged ≥65 years with hip fracture increased from 33,420 in 2021 to 41,994 in 2025, while total healthcare expenditures increased from 205.5 billion to 311.6 billion Korean won (KRW) during the same period (Figure 1) [1]. Despite advances in surgical techniques, perioperative management, and multidisciplinary care, many patients experience postoperative complications, fail to regain their pre-fracture functional status, or die within the first year after injury [2,3,4].

Vitamin D has attracted considerable attention in this population because of its essential roles in both skeletal health and neuromuscular function. Low serum 25-hydroxyvitamin D [25(OH)D] may impair calcium homeostasis through secondary hyperparathyroidism, accelerate bone loss, and contribute to muscle weakness, impaired balance, and increased susceptibility to falls [5,6,7]. Observational studies have reported that low 25(OH)D concentrations are associated with postoperative complications, mortality, impaired functional recovery, poorer quality of life, and subsequent fragility fractures after hip fracture surgery [2,3,4,8,9]. However, vitamin D deficiency frequently coexists with frailty, malnutrition, sarcopenia, multimorbidity, cognitive impairment, and reduced mobility, raising the possibility that low vitamin D status represents a marker of overall vulnerability rather than an isolated causal determinant of adverse outcomes.

The clinical significance of these associations therefore remains uncertain. Published studies have used heterogeneous definitions of vitamin D deficiency, enrolled diverse patient populations, evaluated different clinical outcomes over varying follow-up periods, and differed substantially in adjustment for frailty, nutritional status, and comorbidity. Consequently, the prognostic value of vitamin D may differ according to the severity of deficiency and the outcome evaluated, and observational associations cannot be assumed to demonstrate a clinical benefit from vitamin D supplementation. Likewise, evidence from community-dwelling older adults may not be directly applicable to frail patients recovering from hip fracture [8,9,10].

Therefore, this narrative review aims to critically evaluate the biological and clinical significance of vitamin D deficiency in patients with hip fracture. Specifically, we synthesize current evidence regarding the associations between perioperative vitamin D status and postoperative complications, mortality, functional recovery, quality of life, falls, and secondary fracture; distinguish observational prognostic evidence from evidence evaluating vitamin D supplementation; and discuss the role of vitamin D assessment and correction within comprehensive multidisciplinary post-fracture care.

## 2. Methods

This narrative review was conducted to summarize current evidence on vitamin D deficiency in patients with hip fracture, with a particular focus on postoperative complications, mortality, functional recovery, and vitamin D supplementation strategies. The review was intended to provide a critical synthesis of the available literature rather than a systematic review or meta-analysis.

The initial literature search was conducted during May 2026 and was completed before the targeted supplementary searches performed on 18 July 2026. Relevant studies were identified through PubMed searches and manual screening of the reference lists of key articles. The PubMed search used combinations of terms related to hip fracture and vitamin D, including “hip fracture,” “femoral neck fracture,” “intertrochanteric fracture,” “vitamin D deficiency,” “25-hydroxyvitamin D,” “25(OH)D,” “postoperative complications,” “mortality,” “functional recovery,” “quality of life,” “sarcopenia,” “muscle function,” and “vitamin D supplementation.”

Cohort studies, randomized controlled trials, systematic reviews, meta-analyses, and clinical guidelines were considered when they addressed vitamin D status, vitamin D supplementation, hip fracture outcomes, fracture prevention, or postoperative recovery in older adults. Animal-only studies, basic science investigations without direct clinical relevance, and articles not directly related to hip fracture, vitamin D status, or fracture prevention were excluded.

Because the available studies differed substantially in study design, patient populations, vitamin D cutoff values, intervention strategies, and outcome definitions, findings were synthesized narratively rather than quantitatively pooled. Evidence was interpreted according to study design, consistency of findings, clinical relevance, and the potential influence of confounding.

To strengthen under-referenced sections, four targeted supplementary PubMed searches were conducted on 18 July 2026, without publication-date restrictions; the complete Boolean strategies and search-specific yields are provided in Supplementary Table S1. This targeted evidence-identification exercise was intended to identify additional evidence for narrative synthesis rather than to perform an exhaustive systematic review. The searches retrieved 412 records. After removal of 163 duplicates, 249 records underwent title and abstract screening, and 58 reports were sought for retrieval. Four reports could not be retrieved, and five were excluded at the report stage (three for eligibility and two as duplicate reports), leaving 49 reports for narrative consideration. Of these, five had already been cited in the manuscript, 14 were newly added after verification of the relevant full-text sections, and 30 were retained as relevant background evidence but were not cited separately because citation selection prioritized direct relevance to the targeted evidence gaps, balance between positive and null or inconsistent findings, and avoidance of overlapping cohorts or redundant evidence. Independent duplicate screening and formal risk-of-bias assessment were not performed.

## 3. Interpretation of Vitamin D Status in Patients with Hip Fracture

Vitamin D is derived from cutaneous synthesis, dietary intake, and supplementation. It is first hydroxylated in the liver to 25-hydroxyvitamin D [25(OH)D], the major circulating form, and subsequently converted primarily in the kidney to the biologically active metabolite, 1,25-dihydroxyvitamin D [1,25(OH)_2_D]. Serum 25(OH)D is the standard biomarker used to assess vitamin D status because it reflects vitamin D derived from sunlight exposure, diet, and supplementation. In contrast, circulating 1,25(OH)_2_D is tightly regulated by parathyroid hormone, calcium, and phosphate concentrations and may remain normal or even increase despite depleted vitamin D stores. For this reason, serum 25(OH)D, rather than 1,25(OH)_2_D, is regarded as the most appropriate indicator of vitamin D status [5].

The definition of vitamin D deficiency varies substantially across studies. Most studies involving patients with hip fracture have defined deficiency as a serum 25(OH)D concentration < 20 ng/mL (50 nmol/L) and insufficiency as 20–30 ng/mL. Other investigators have used a broader threshold of <30 ng/mL to define low vitamin D status, whereas studies focusing on more severe depletion have adopted cutoff values of 10–12 ng/mL. The 2024 Endocrine Society guideline concluded that current evidence is insufficient to establish universal serum 25(OH)D thresholds for sufficiency, insufficiency, or deficiency in the general population [10]. Accordingly, the cutoff values used in hip fracture studies should be regarded as study-specific operational definitions rather than universally accepted diagnostic categories. For reference, 1 ng/mL is equivalent to approximately 2.5 nmol/L.

Importantly, a threshold of <30 ng/mL encompasses both mild insufficiency and more pronounced deficiency, whereas concentrations of 10–12 ng/mL identify a subgroup with more severe depletion. Consequently, findings from studies using different cutoff values are not directly comparable, and the prognostic significance of vitamin D deficiency should be interpreted in the context of the threshold applied.

Low serum 25(OH)D frequently coexists with malnutrition, frailty, sarcopenia, limited mobility, renal dysfunction, comorbidity, and disturbances in the calcium–parathyroid hormone axis. Serum 25(OH)D should therefore be interpreted as both a potentially modifiable metabolic abnormality and a marker of systemic vulnerability rather than as an isolated causal determinant of adverse outcomes. Throughout this review, individual studies are interpreted according to their vitamin D cutoff values and the specific clinical outcomes evaluated.

## 4. Bone–Muscle–Frailty Pathways (Figure 2)

### 4.1. Skeletal Pathway

Vitamin D contributes to skeletal health by regulating calcium and phosphate homeostasis, promoting intestinal calcium absorption, and supporting bone mineralization. Vitamin D deficiency reduces calcium absorption, stimulates secondary hyperparathyroidism, and increases bone resorption, thereby contributing to skeletal fragility [5]. In older adults with hip fracture, vitamin D deficiency frequently coexists with osteoporosis, inadequate calcium intake, malnutrition, and age-related impairment of bone remodeling.

These mechanisms may adversely influence postoperative recovery, particularly fracture healing after internal fixation. However, current clinical evidence does not establish vitamin D deficiency as an independent cause of delayed union, nonunion, implant failure, or periprosthetic fracture, and its clinical impact likely varies according to fracture type, surgical treatment, bone quality, and secondary hyperparathyroidism [4,5].

### 4.2. Muscle and Physical-Performance Pathway

Vitamin D may influence both hip fracture susceptibility and postoperative recovery through its effects on skeletal muscle and neuromuscular function. Vitamin D receptors are expressed in skeletal muscle, and vitamin D signaling has been associated with muscle strength, balance, and physical performance [5,6,7]. Accordingly, low serum 25-hydroxyvitamin D [25(OH)D] may contribute to falls and impaired rehabilitation after hip fracture, although evidence linking vitamin D deficiency directly to sarcopenia remains inconsistent.

Kim et al. [6] reported that patients undergoing hip fracture surgery had significantly lower serum 25(OH)D concentrations and poorer handgrip strength than patients undergoing elective total hip arthroplasty. However, sarcopenia prevalence did not differ significantly between groups, and vitamin D levels were not associated with sarcopenia within the hip fracture cohort, suggesting that low 25(OH)D is more closely related to impaired muscle performance than to sarcopenia itself.

Wang et al. [11] demonstrated that both femoral-neck bone mineral density and the pectoralis muscle index partially mediated the association between vitamin D status and hip fracture risk, with a greater contribution from muscle-related measures. However, the retrospective design precludes causal inference. Similarly, Skúladóttir et al. [7] showed that low serum 25(OH)D was associated with lower bone density, reduced leg strength, impaired balance, and an increased risk of incident hip fracture. Adjustment for bone and physical function attenuated this association, suggesting that both skeletal and muscle-related pathways contribute to fracture risk.

Overall, available evidence supports an interaction between low vitamin D, impaired muscle function, and skeletal fragility. However, most studies have evaluated hip fracture occurrence rather than postoperative recovery, and direct evidence that vitamin D supplementation improves muscle recovery after hip fracture remains limited.

### 4.3. Frailty and Systemic Vulnerability

Low serum 25(OH)D in patients with hip fracture frequently coexists with frailty, malnutrition, sarcopenia, limited mobility, renal dysfunction, chronic illness, and comorbidity [4,5,6,7,11]. These factors may independently increase postoperative complications and mortality while also contributing to lower vitamin D concentrations.

Because serum 25(OH)D is usually measured at hospital admission after fracture, low levels may reflect not only vitamin D deficiency but also pre-fracture functional decline, nutritional status, reduced sunlight exposure, and overall health. Even after statistical adjustment, residual confounding by frailty and functional status is difficult to eliminate.

**Figure 2:**
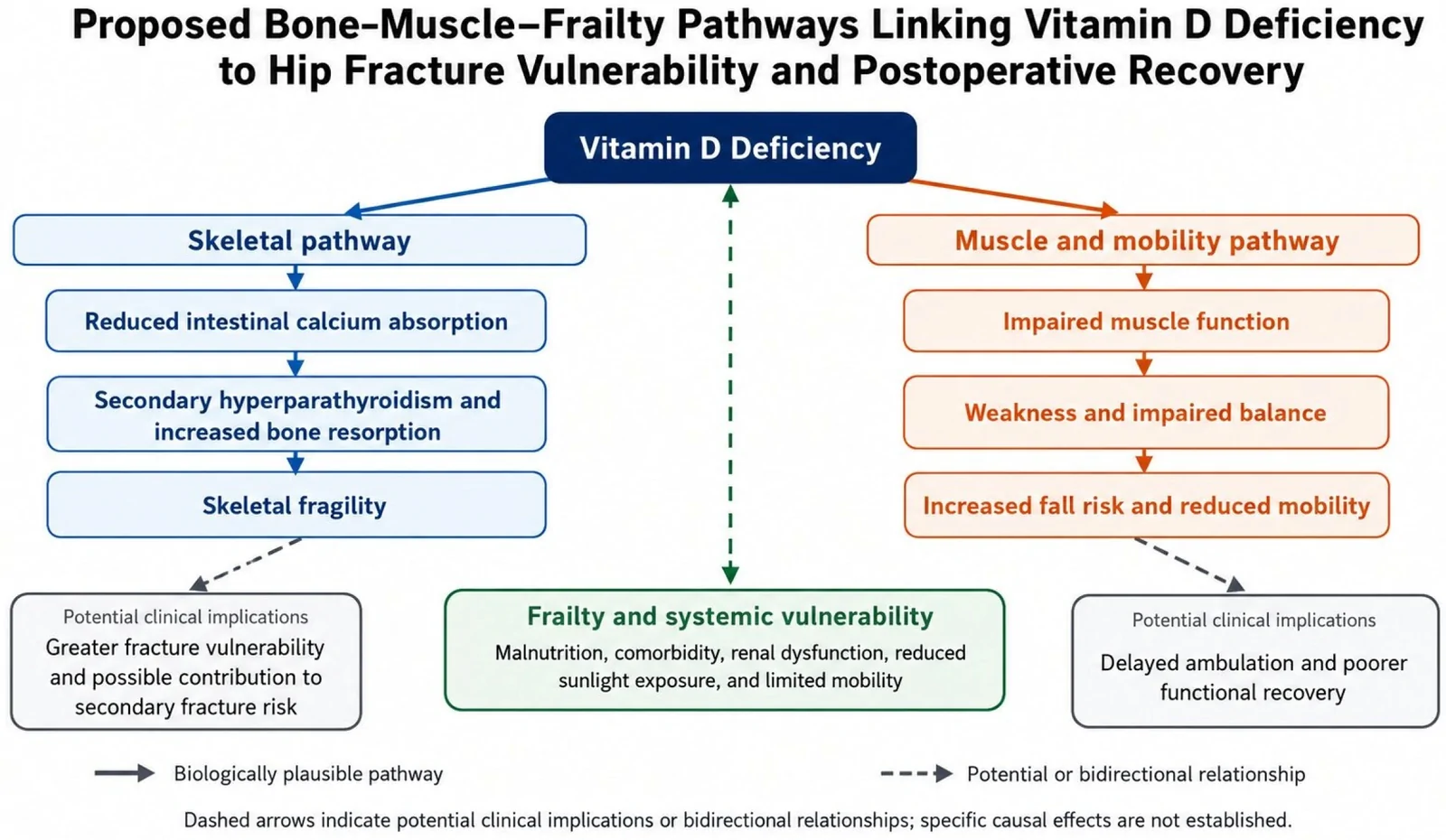
Proposed bone–muscle–frailty pathways linking vitamin D deficiency to hip fracture vulnerability and postoperative recovery. Solid arrows indicate biologically plausible musculoskeletal pathways, whereas dashed arrows indicate potential clinical implications or bidirectional relationships. The figure should not be interpreted as establishing causal effects of vitamin D deficiency on specific postoperative outcomes.

Accordingly, vitamin D deficiency should be interpreted as both a potentially modifiable metabolic abnormality and a marker of systemic vulnerability rather than as an isolated determinant of adverse outcomes. A bone–muscle–frailty framework may therefore better explain the observed associations between low vitamin D status and hip fracture susceptibility, postoperative complications, and functional recovery (Figure 2) [2,3,4,5,6,7,8,9,11].

## 5. Clinical Outcomes Associated with Vitamin D Status After Hip Fracture (Table 1)

### 5.1. Postoperative Complications

Several observational studies have reported associations between low perioperative serum 25(OH)D concentrations and selected postoperative complications after hip fracture surgery (Table 1). The most consistent associations have been observed for delirium, pneumonia, and short-term medical readmission, whereas thromboembolic events, composite medical complications, surgical-site infection, nonunion, implant failure, and other orthopaedic complications have shown inconsistent or absent associations [2,3,4,12].

Differences among studies likely reflect variation in vitamin D thresholds, timing of blood sampling, outcome definitions, and adjustment for frailty and comorbidity. Secondary hyperparathyroidism may further identify patients with greater metabolic vulnerability rather than directly explaining postoperative complications [12].

Overall, current evidence suggests that low perioperative 25(OH)D is associated with selected early medical complications but not consistently with orthopaedic complications or long-term adverse events. Residual confounding by frailty, nutritional status, cognition, and comorbidity limits causal interpretation [2,3,4,12].

### 5.2. Mortality

Multiple cohort studies and recent meta-analyses have evaluated the association between vitamin D deficiency and mortality after hip fracture (Table 1). Overall, frank vitamin D deficiency (<20 ng/mL) has been associated with increased mortality, whereas mild insufficiency has shown less consistent prognostic significance [4,8,13,14,15,16].

**Table 1:** Observational Evidence on Vitamin D Status and Clinical Outcomes After Hip Fracture.

Study	Design and Participants	25(OH)D Assessment	Outcomes and Follow-Up	Principal Findings	Key Interpretation and Limitations
Fink et al. [2].	Retrospective single-center cohort; *n* = 314 surgically treated low-energy proximal femoral fractures	Preoperative; <30 vs. ≥30 ng/mL; additional subgroup < 10 ng/mL	Early postoperative complications within 30 days; late complications from 30 days to 1 year	Early complications occurred in 34.2% vs. 20.6%; age- and sex-adjusted OR, 2.06 (95% CI, 1.14–3.73). Late complications were not associated with vitamin D status: OR, 1.08 (95% CI, 0.40–2.95). The <10-ng/mL subgroup had higher crude early and overall complication rates.	Broad < 30-ng/mL threshold; limited adjustment; heterogeneous complications; no adjusted severity gradient.
Lim et al. [3].	Retrospective single-center cohort; *n* = 1029 patients aged >65 years undergoing hip fracture surgery	On admission before surgery; <20 vs. ≥20 ng/mL	In-hospital delirium, pneumonia, thromboembolism, and surgical-site infection; length of stay; postoperative Koval mobility score at final follow-up	Deficiency was associated with delirium: OR, 1.52 (95% CI, 1.01–2.31), and pneumonia: OR, 3.82 (95% CI, 1.29–11.34). Thromboembolism was not significant: OR, 2.41 (95% CI, 0.79–7.34). LOS was 27.7 ± 17.8 vs. 20.9 ± 11.8 days: OR, 1.03 (95% CI, 1.02–1.05). Postoperative Koval score was 4.0 ± 2.1 vs. 3.1 ± 1.9: OR, 1.21 (95% CI, 1.11–1.32).	Retrospective single-center study with nonuniform follow-up; admission sampling and unmeasured PTH, sarcopenia, rehabilitation, and post-discharge factors limit inference.
Ingstad et al. [4].	Hip-fracture registry with retrospective outcome review; 872 fractures in 865 patients	First or second postoperative day; <50 vs. ≥50 nmol/L	Delirium; acute medical complications; medical readmission at 30 days, 12 weeks, and 1 year; orthopedic complications, secondary surgery, mortality, and contralateral hip fracture within 1 year	Deficiency was associated with delirium, 30-day and 12-week medical readmission, and contralateral hip fracture within 1 year (OR, 2.84; 95% CI, 1.15–7.03), but not with 1-year medical readmission, orthopaedic complications, secondary surgery, or mortality.	Postoperative sampling and retrospective follow-up; multiple comparisons; BMI and supplementation adherence unavailable.
Wang et al. [8].	Systematic review and random-effects meta-analysis of 18 cohort studies; *n* = 10,237	Study-specific measurements; deficiency < 20 ng/mL, insufficiency 20–30 ng/mL, and sufficient category > 30 ng/mL	All-cause mortality over study-specific follow-up periods	Deficiency was associated with mortality: HR, 2.29 (95% CI, 1.41–3.72; I^2^ = 25.4%). Insufficiency was not significant: HR, 1.10 (95% CI, 0.97–1.24; I^2^ = 72.2%). The >30-ng/mL category was also not significant: HR, 1.04 (95% CI, 0.92–1.18; I^2^ = 73.8%).	Heterogeneous definitions, assays, follow-up, and adjustment; publication bias; trim-and-fill affected the overall estimate.
Llombart et al. [9].	Systematic review and meta-analysis of 7 observational cohorts; *n* = 1972	Primarily deficiency < 20 ng/mL; one included study used ≤ 22.5 nmol/L (9 ng/mL)	Walking ability, hospital length of stay, and combined functional ability or QOL at study-specific follow-up	Walking ability was not significantly different: OR, 0.68 (95% CI, 0.31–1.53; I^2^ = 69%); adjusted analysis: OR, 0.60 (95% CI, 0.25–1.42). LOS was not significantly different: MD, 2.27 days (95% CI, −2.47 to 7.01; I^2^ = 93%). Deficiency was associated with worse functional ability or QOL: SMD, −1.50 (95% CI, −2.88 to −0.12; I^2^ = 96%).	Only three studies per pooled outcome; high heterogeneity and possible publication bias.
Alarcón et al. [12].	Cross-sectional single-center orthogeriatric cohort; *n* = 607 adults aged >64 years with acute fragility hip fracture	During the first 5 hospital days, mean 3.3 days after admission; severe deficiency < 12 ng/mL, moderate deficiency 12–20 ng/mL; SHPT defined as PTH > 65 pg/mL	Active medical problems requiring treatment during hospitalization; PTH response within each deficiency stratum	SHPT occurred in 45.5% of patients with <12 ng/mL and 28.0% with 12–20 ng/mL. Each additional medical problem was associated with greater odds of SHPT in the severe-deficiency group: OR, 1.175 (95% CI, 1.073–1.288), and moderate-deficiency group: OR, 1.289 (95% CI, 1.101–1.510). Lower 25(OH)D and poorer eGFR were also associated with SHPT in severe deficiency.	Cross-sectional design precludes causal inference; post-fracture sampling and unmeasured calcium, magnesium, and other PTH determinants.
Bayram et al. [13].	Retrospective single-center cohort; *n* = 1510 patients aged >50 years	On ward admission; <50 vs. ≥50 nmol/L	All-cause mortality over a median 405 days; acute hospital length of stay	During follow-up, 464 patients died. Deficiency was independently associated with mortality: HR, 1.26 (95% CI, 1.03–1.53). One-year survival was 71.7% vs. 79.0%, and 2-year survival was 56.7% vs. 67.4%. LOS did not differ between groups.	Vitamin D was missing in 565 eligible patients; limited comorbidity and BMI data and residual confounding.
Llombart et al. [14].	Systematic review and meta-analysis of mortality after hip fracture	Study-specific peri-fracture 25(OH)D measurements and deficiency definitions	All-cause mortality over study-specific follow-up periods	Associations apparent in unadjusted analyses were no longer statistically significant when adjusted effect estimates were used.	Associations attenuated after adjustment, indicating sensitivity to frailty and comorbidity.
Ho et al. [15].	Large propensity-matched cohort of patients undergoing hip fracture surgery	Deficiency ≤ 20 ng/mL; insufficiency 20–30 ng/mL	30-day, 90-day, and 12-month mortality	Deficiency was associated with higher short- and long-term mortality, whereas insufficiency was not associated with mortality (HR, 1.09; 95% CI, 0.97–1.22).	Propensity matching reduced measured imbalance but cannot eliminate residual confounding or establish causality.
Lee et al. [16].	Retrospective review of prospectively collected data; *n* = 489 patients aged ≥50 years	At admission; severe deficiency < 10 ng/mL, deficiency 10–20 ng/mL, insufficiency 20–30 ng/mL, normal ≥ 30 ng/mL	Mortality over a minimum follow-up of 1 year	Vitamin D deficiency was associated with mortality in univariate analysis (*p* = 0.012) but was not independently associated in the Cox model. Independent predictors were ASA score: HR, 12.220 (95% CI, 3.314–45.063), and poorer pre-fracture ambulatory status: HR, 1.681 (95% CI, 1.104–2.558).	Association attenuated after adjustment; retrospective design, one measurement, and inconsistent deficient-patient counts.
Sim et al. [17].	Retrospective single-center cohort; *n* = 664 surgically treated fragility hip fractures	On admission; severe deficiency ≤ 10 ng/mL, mild deficiency 10–20 ng/mL, insufficiency 20–30 ng/mL, normal > 30 ng/mL	Parker Mobility Score, Harris Hip Score, and SF-36 domains at baseline and 6 months	Using severe deficiency as reference, adjusted 6-month Parker Mobility Scores were higher in the normal, insufficient, and mildly deficient groups by 0.81 (95% CI, 0.06–1.55), 0.67 (0.05–1.28), and 0.72 (0.11–1.34) points, respectively. Normal status was associated with an 8.75-point higher SF-36 Physical Functioning score (95% CI, 0.45–17.04); intermediate categories were not significant for SF-36 PF.	Baseline function differed; retrospective data did not assess supplementation effects.
Hao et al. [18].	Prospective secondary analysis of a 47-site trial cohort; *n* = 290	Preoperative; <12, 12–20, 20–30, and ≥30 ng/mL	Ability to walk 10 feet without human assistance and mortality at 30 and 60 days	Compared with <12 ng/mL, adjusted ORs for walking at 30 days were 2.61 (95% CI, 1.13–5.99), 3.48 (1.53–7.95), and 2.84 (1.12–7.20) across the three higher categories. At 60 days, corresponding ORs were 2.67 (1.14–6.25), 3.42 (1.46–8.00), and 3.67 (1.37–9.82). Vitamin D was not associated with 30- or 60-day mortality.	Restricted secondary analysis with mainly age and sex adjustment; findings suggest a severe-deficiency threshold, not a linear dose response.
Gumieiro et al. [19].	Prospective single-center observational cohort; *n* = 100 analyzed after exclusion of two pathological fractures	Serum 25(OH)D measured within 72 h of admission	Concurrent handgrip strength, MUAMC, and hospital length of stay	After adjustment for age and sex, serum 25(OH)D was associated with handgrip strength but not with MUAMC or LOS.	Concurrent measurements support association with strength, not longitudinal recovery.
Cappola et al. [20].	Prospective longitudinal cohort from eight hospitals; biomarker analysis *n* = 305 (156 men, 149 women)	During admission and at 2, 6, and 12 months; analyzed continuously	SPPB at 2, 6, and 12 months during the first postoperative year	In the combined adjusted analysis, each 10-ng/mL higher 25(OH)D was associated with a 0.26-point higher SPPB score (95% CI, 0.01–0.51). The association did not significantly differ by sex (interaction *p* = 0.54).	Effect below common clinically meaningful SPPB thresholds; high attrition and limited diversity.
Ren et al. [21].	Systematic review and meta-analysis of 19 cohort studies of older adults after hip fracture	Study-specific assessment; risk factor reported as combined calcium/vitamin D deficiency rather than vitamin D deficiency alone	Incidence and risk factors for secondary hip fracture over study-specific follow-up periods	Pooled secondary hip-fracture incidence: 10.29% (95% CI, 9.7–10.9%); women: 14.94% (95% CI, 12.3–17.8%); men: 9.89% (95% CI, 8.3–11.6%). Calcium/vitamin D deficiency was identified as a significant risk factor with moderate-certainty evidence; PAF, 1.12% (95% CI, 0.7–1.6%).	Calcium and vitamin D deficiency were combined; PAF does not establish treatment efficacy.

The individual functional studies by Hao et al., Gumieiro et al. and Lim et al. were also included in the Llombart et al. meta-analysis. They should therefore not be interpreted as independent replications when discussing the total amount of evidence [3,9,18,19]. The individual secondary-fracture study by Ingstad et al. was not included in the Ren et al. meta-analysis and therefore does not represent duplicate evidence [4,21]. Abbreviations: 25(OH)D, 25-hydroxyvitamin D; ASA, American Society of Anesthesiologists; BMI, body mass index; CI, confidence interval; eGFR, estimated glomerular filtration rate; HR, hazard ratio; LOS, length of stay; MD, mean difference; MUAMC, mid-upper arm muscle circumference; OR, odds ratio; PTH, parathyroid hormone; QOL, quality of life; SF-36 PF, 36-Item Short Form Health Survey Physical Functioning; SHPT, secondary hyperparathyroidism; SMD, standardized mean difference; SPPB, Short Physical Performance Battery.

However, the observed association is sensitive to study design and statistical adjustment. Meta-analyses have demonstrated heterogeneity, possible publication bias, and substantial variation in vitamin D thresholds, follow-up duration, and adjustment for confounding factors [8]. Adjusted findings were inconsistent across studies: some reported no independent association with mortality, whereas others found significant associations after multivariable adjustment or propensity-score matching [4,13,15,16]. A meta-analysis likewise found no significant association after adjustment for potential confounders [14].

Taken together, current evidence supports low serum 25(OH)D as a prognostic marker of mortality after hip fracture rather than a confirmed independent cause of death.

### 5.3. Functional Recovery and Quality of Life

Functional recovery has been evaluated using diverse outcome measures, including independent walking, mobility scales, physical performance, handgrip strength, activities of daily living, hospital length of stay, and health-related quality of life (Table 1). Because these outcomes reflect different aspects of recovery, direct comparison across studies is limited [3,4,9,13,16,17,18,19,20].

The most consistent findings have been observed in patients with severe vitamin D deficiency (approximately ≤10–12 ng/mL), who demonstrated poorer early walking recovery and lower postoperative mobility scores [17,18]. In contrast, studies using broader deficiency thresholds reported variable associations with walking ability, hospital stay, handgrip strength, and quality of life [3,9,19,20].

Overall, severe vitamin D deficiency appears to be associated with impaired postoperative mobility, whereas evidence for milder deficiency remains inconsistent. Recovery after hip fracture is also strongly influenced by pre-fracture functional status, cognitive impairment, nutritional status, rehabilitation intensity, and comorbidity [3,9,17,18,19,20].

### 5.4. Falls and Secondary Hip Fracture

Evidence linking perioperative vitamin D status with recurrent falls after hip fracture remains limited (Table 1). Most available studies have instead evaluated contralateral or secondary hip fracture [4,7].

One cohort study demonstrated an association between vitamin D deficiency and contralateral hip fracture during the first postoperative year, although the number of events was small and residual confounding could not be excluded [4]. A subsequent meta-analysis identified combined calcium/vitamin D deficiency as one of several risk factors for secondary hip fracture, highlighting the multifactorial nature of recurrent fractures [21].

Overall, low vitamin D status may identify patients at increased risk of secondary hip fracture, although its independent contribution cannot be distinguished reliably from osteoporosis, impaired muscle function, frailty, nutritional status, and cognitive dysfunction [4,21].

## 6. Sources of Heterogeneity Across Studies

Reported associations vary because studies differ in vitamin D definitions and measurement, outcomes, populations, follow-up, care pathways, and adjustment for frailty. These differences must be considered before 25(OH)D is interpreted as either an independent prognostic factor or a clinically irrelevant measurement.

### 6.1. Variation in Vitamin D Thresholds

Studies defined low vitamin D as <30 ng/mL, <20 ng/mL, <50 nmol/L, or severe deficiency at approximately 10–12 ng/mL. These thresholds represent different populations: <30 ng/mL includes mild insufficiency, whereas 10–12 ng/mL identifies marked depletion [2,3,4,8,9,10,12,13,16,17,18].

Associations varied accordingly. Fink et al. linked <30 ng/mL with early complications, Wang et al. linked <20 ng/mL—but not 20–30 ng/mL—with mortality, and Hao and Sim reported the clearest mobility associations below approximately 10–12 ng/mL [2,8,17,18].

Marked deficiency may be more informative than mild insufficiency for selected outcomes, but the studies used different thresholds, reference groups, outcomes, and baseline-risk populations. The apparent severity-specific pattern is therefore hypothesis-generating rather than proof of a continuous dose–response relationship [8,9,10,17,18].

### 6.2. Timing and Method of 25(OH)D Measurement

Sampling ranged from admission before surgery to the first postoperative days. Postoperative concentrations may be influenced by fluids, inflammation, blood loss, nutritional interruption, and acute stress; Hao et al. observed an early postoperative decline, whereas Ingstad et al. sampled on postoperative days 1–2, limiting direct comparability [4,18].

A single peri-fracture measurement cannot distinguish chronic depletion from acute change, and the Baltimore Hip Studies showed that 25(OH)D varied during the following year. Differences in assay calibration may further misclassify values near categorical thresholds [3,9,18,20].

### 6.3. Heterogeneity in Outcomes and Follow-Up

Outcomes varied considerably in both timing and clinical meaning. Complication studies evaluated individual events, composite outcomes, readmissions, and orthopaedic complications, whereas recovery studies assessed walking ability, mobility scales, physical-performance tests, handgrip strength, length of hospital stay, and quality of life [2,3,4,8,9,13,16,17,18,19,20,21].

These outcomes are not interchangeable because they assess different aspects of recovery. For example, independent walking reflects an early functional milestone, whereas mobility scales and physical-performance tests evaluate broader domains of function. Accordingly, an association observed for one outcome should not be assumed to apply to others.

This heterogeneity was evident in the meta-analysis by Llombart et al., in which vitamin D deficiency was associated with poorer functional ability or quality of life but not with walking ability or hospital stay [9]. Similarly, Fink et al. and Ingstad et al. reported associations with early postoperative complications or short-term readmission, whereas longer-term outcomes were largely unaffected [2,4]. As follow-up length increases, rehabilitation, living environment, treatment adherence, and subsequent medical events may exert a greater influence on recovery than baseline vitamin D status.

### 6.4. Differences in Patient Populations and Care Pathways

Populations differed in age, sex, residence, comorbidity, cognition, pre-fracture mobility, nutrition, and fracture type, affecting both vitamin D status and outcome risk [2,3,4,13,16,17,18,19,20].

In the Baltimore Hip Studies, men had lower 25(OH)D than women throughout the first postoperative year, and bone-density associations differed by sex, although physical-performance associations did not show a significant interaction [20].

Regional subgroup findings may reflect limited study numbers and differences in selection, supplementation, orthogeriatric services, rehabilitation, and post-discharge management rather than geographic biology [8].

Care pathways also varied in supplementation, loading doses, osteoporosis treatment, adherence, rehabilitation, and social support, complicating attribution to baseline 25(OH)D [4,20].

### 6.5. Confounding and Statistical Adjustment

Vitamin D deficiency frequently coexists with reduced mobility, malnutrition, low body mass index, sarcopenia, cognitive impairment, renal dysfunction, chronic illness, institutional residence, and limited sunlight exposure, all of which are independently associated with poor outcomes after hip fracture [2,3,4,6,12,13,16,17,18,19,20].

The extent of statistical adjustment varied substantially across studies, ranging from models including only age and sex to multivariable analyses incorporating body mass index, bone density, baseline functional status, anemia, comorbidity, and pre-fracture care [2,3,4,13,16,17]. Consequently, associations reported in minimally adjusted analyses were often attenuated or lost after adjustment for frailty-related variables [4,13,16].

Furthermore, several variables—including muscle weakness, nutritional status, and mobility—may act as both confounders and mediators. Therefore, both inadequate adjustment and overadjustment may influence observed associations, highlighting the need for prospective studies using repeated measurements and prespecified causal models.

### 6.6. Study Design, Publication Bias, and Interpretation

Most available evidence is derived from retrospective cohorts, registry studies, secondary analyses, or cross-sectional investigations, all of which are susceptible to missing data, selection bias, retrospective outcome ascertainment, and loss to follow-up [2,3,4,8,9,12,13,16,17,18,19,20,21].

Meta-analysis cannot fully overcome these limitations. Publication bias attenuated the pooled mortality estimate after trim-and-fill analysis, and the functional meta-analysis demonstrated funnel-plot asymmetry together with sensitivity to high-weight studies [8,9].

Overall, the available evidence supports an outcome-specific and severity-aware interpretation of vitamin D status. Severe deficiency appears more clinically informative than mild insufficiency for selected outcomes; however, associations remain influenced by differences in study design, vitamin D thresholds, patient populations, sampling methods, and statistical adjustment. Low serum 25(OH)D is therefore best regarded as one component of a bone–muscle–frailty phenotype rather than as a uniform cause of adverse outcomes [2,3,4,8,9,13,16,17,18,19,20,21].

## 7. Vitamin D Supplementation and Post-Fracture Management (Table 2)

Associations between low serum 25-hydroxyvitamin D [25(OH)D] and adverse clinical outcomes should be distinguished from evidence evaluating the effects of vitamin D supplementation. Although supplementation consistently increases serum 25(OH)D concentrations, higher or loading-dose regimens have not consistently improved postoperative mobility, complications, mortality, or secondary fracture outcomes after hip fracture [4,22,23,24,25,26,27,28,29,30].

**Table 2:** Intervention and Care-Pathway Evidence for Vitamin D Management After Hip Fracture.

Study	Design and Participants	Intervention and Comparator	Outcomes and Follow-Up	Principal Findings	Key Interpretation and Limitations
Cuadra-Llopart et al. [22]	Randomized, controlled, parallel-group, single-blind trial; *n* = 50 adults aged ≥75 years with hip fracture and 25(OH)D < 30 ng/mL	Calcifediol 266 μg daily for 5 days vs. 266 μg weekly for 5 weeks, followed by 25(OH)D-guided maintenance	Serum 25(OH)D, PTH, and calcium; Barthel Index and absolute or relative functional gain at discharge and 3, 6, and 12 months	At 1 month, 25(OH)D was 38.2 vs. 35.2 ng/mL (*p* = 0.50); PTH was 43.5 vs. 75.2 pg/mL (*p* = 0.001). About half achieved clinically meaningful functional improvement; long-term functional outcomes did not differ significantly. No increased hypercalcemia risk was observed.	Both active regimens corrected deficiency; the small trial had no placebo group and did not demonstrate long-term functional superiority of daily loading.
LeBoff et al.—VITAL ancillary study [23]	Randomized placebo-controlled trial; *n* = 25,871 generally healthy midlife and older adults	Vitamin D_3_ 2000 IU/day vs. placebo	Total, nonvertebral, and hip fractures over median 5.3 years	No reduction in total fracture: HR, 0.98; nonvertebral fracture: HR, 0.97; or hip fracture: HR, 1.01	Few participants were severely deficient or osteoporotic, limiting applicability to frail patients after hip fracture.
Zhao et al. [24].	Systematic review and meta-analysis of 33 randomized trials; *n* = 51,145 community-dwelling adults > 50 years	Calcium, vitamin D, or combined supplementation vs. placebo or no treatment	Hip, nonvertebral, vertebral, and total fractures	No significant fracture reduction with calcium, vitamin D, or their combination; combined calcium/vitamin D hip-fracture RR, 1.09 (95% CI, 0.85–1.39)	Community-dwelling populations do not represent severely deficient, institutionalized, or recent hip-fracture patients.
Avenell et al. [25].	Cochrane systematic review of randomized trials in postmenopausal women and older men	Vitamin D or analogues, alone or combined with calcium	Hip, nonvertebral, vertebral, and any fracture; adverse events	Vitamin D alone was unlikely to prevent fracture; vitamin D plus calcium produced small benefits in selected analyses, particularly in institutional settings	Heterogeneous regimens and adherence limit interpretation; calcium-containing regimens may increase adverse effects.
Yao et al. [26].	Systematic review and meta-analysis of observational studies and randomized trials	Vitamin D alone or vitamin D plus calcium vs. control	Any fracture and hip fracture	Vitamin D alone did not reduce any fracture or hip fracture. Combined vitamin D and calcium produced modest reductions in any fracture and hip fracture	Broader prevention populations and heterogeneous regimens limit applicability to post-fracture rehabilitation.
Mak et al. [27].	Randomized, double-blind, placebo-controlled trial; *n* = 218 adults aged ≥65 years after hip fracture surgery	Single 250,000-IU vitamin D_3_ loading dose vs. placebo; both groups received vitamin D 800 IU and calcium 500 mg daily for 26 weeks	Serum 25(OH)D, 2.4-m gait velocity, falls, fractures, pain, QOL, and mortality up to 26 weeks	Loading increased 25(OH)D at weeks 2 and 4, but not week 26. Gait velocity did not differ at week 4. Falls were 6.3% vs. 21.1% (*p* = 0.024); fractures and mortality did not differ significantly	The trial was not powered for falls, fractures, or mortality; both groups received maintenance vitamin D and calcium. Biochemical improvement did not translate into gait improvement.
Stemmle et al. [28].	Secondary analysis of a factorial randomized clinical trial; *n* = 173; mean age 84 years	Vitamin D_3_ 800 vs. 2000 IU daily, with a simple home exercise program or standard physiotherapy alone	Timed Up and Go, knee-flexor and extensor strength, and PF-10 at baseline, 6, and 12 months	Significant vitamin D dose–exercise interaction for TUG (*p* = 0.045). The 800-IU plus exercise group performed better than the 800-IU without-exercise group at 12 months (13.8 vs. 19.5 s; *p* = 0.010). No treatment differences in knee-extensor strength or PF-10	The study did not establish superiority of 2000 IU; benefit occurred with 800 IU plus exercise. Secondary analysis, attrition, and incomplete exercise adherence limit inference.
Torbergsen et al. [29].	Preplanned substudy of a randomized orthogeriatric-care trial in older patients after hip fracture	Orthogeriatric care with individualized nutrition advice plus vitamin K1, calcium, vitamin D, and other nutritional supplements versus usual orthopedic care	Serum 25(OH)D and bone-turnover markers at 4 months	At 4 months, serum 25(OH)D concentrations were higher in the intervention group than in the control group, whereas bone-turnover markers did not differ between groups.	Because the intervention was multicomponent, the independent effect of vitamin D could not be determined.
Ekinci et al. [30].	Randomized controlled study; *n* = 75 randomized, *n* = 62 analyzed; malnourished older women with hip fracture	Enteral product containing 3 g CaHMB, 1000 IU vitamin D, and 36 g protein plus standard nutrition vs. standard postoperative nutrition	Wound healing, mobility, muscle strength, anthropometric and laboratory measures through postoperative day 30	Wound healing was shorter; 81.3% vs. 26.7% were mobile on days 15 and 30 (*p* = 0.001); day-30 muscle strength was greater in the intervention group	Benefits cannot be attributed to vitamin D within the multicomponent intervention. The study included only malnourished older women and 30-day follow-up.
Piziak and Rajab [31]	Prospective nonrandomized quality-improvement study; *n* = 137 patients aged >50 years qualifying for osteoporosis medication	Standardized discharge orders offering calcium/vitamin D, DXA, and endocrinology consultation	Treatment uptake and completion of recommended assessment over at least 6 months	Calcium/vitamin D use increased from 37/137 to 92/137; bisphosphonate use increased from 17/137 to 65/137 (both *p* < 0.0001). All were offered the pathway; 82 completed DXA and 54 attended endocrinology	Nonrandomized before-and-after data show improved care processes, not clinical efficacy. Follow-up remained incomplete.

Abbreviations: 25(OH)D, 25-hydroxyvitamin D; CaHMB, calcium β-hydroxy-β-methylbutyrate; CI, confidence interval; DXA, dual-energy X-ray absorptiometry; HR, hazard ratio; PF-10, 10-item Physical Functioning scale; QOL, quality of life; RR, risk ratio; TUG, Timed Up and Go.

### 7.1. Direct Intervention Evidence After Hip Fracture

Randomized and observational studies consistently demonstrate that vitamin D supplementation increases serum 25(OH)D concentrations after hip fracture but has not consistently improved postoperative mobility, complications, mortality, or recurrent fracture outcomes [4,27,28]. Loading-dose regimens produced transient increases in serum 25(OH)D without significant improvements in gait velocity or survival [27]. Similarly, higher daily doses were not superior to standard-dose supplementation, whereas structured exercise combined with standard-dose vitamin D was associated with better functional performance, suggesting that rehabilitation rather than vitamin D dose may explain the observed benefit [28]. Although one nonrandomized study reported fewer early orthopaedic complications after introducing a loading-dose protocol, concurrent changes in osteoporosis management preclude causal interpretation [4]. Cuadra-Llopart et al. randomized 50 patients aged 75 years or older with hip fracture and confirmed vitamin D deficiency [25(OH)D < 30 ng/mL] to calcifediol 266 μg daily for 5 days or 266 μg weekly for 5 weeks, followed by 25(OH)D-guided maintenance therapy. At 1 month, both regimens corrected vitamin D deficiency (mean 25(OH)D, 38.2 vs. 35.2 ng/mL; *p* = 0.50), while PTH was lower with the daily loading regimen (43.5 vs. 75.2 pg/mL; *p* = 0.001). Approximately half of the participants achieved clinically meaningful functional improvement, but long-term Barthel Index and absolute or relative functional gain did not differ significantly between groups through 12 months. Neither regimen increased the risk of hypercalcemia [22].

### 7.2. Multicomponent Nutritional Intervention

Vitamin D has also been evaluated as part of multicomponent nutritional interventions after hip fracture. Combined nutritional strategies incorporating vitamin D together with calcium, protein, vitamin K, or β-hydroxy-β-methylbutyrate improved serum vitamin D concentrations, mobility, muscle strength, wound healing, or biochemical markers in some studies [29,30]. However, because vitamin D was administered with other nutritional components, its independent contribution could not be determined.

### 7.3. Evidence for Fracture Prevention in Broader Populations

Large randomized trials and meta-analyses conducted in community-dwelling older adults have generally shown that vitamin D alone does not reduce fractures, including hip fracture [23,24,25,26]. Combined vitamin D and calcium produced modest fracture reduction in some analyses, particularly among institutionalized older adults, whereas other meta-analyses found no significant benefit [24,25,26]. These findings should be interpreted cautiously because they were derived largely from populations without recent hip fracture or severe vitamin D deficiency and therefore may not be directly applicable to postoperative hip fracture care [23,24,25,26].

### 7.4. Dose, Regimen, and Safety

Current evidence does not support a simple dose-response relationship between higher vitamin D doses and better clinical outcomes. High-dose loading regimens increase serum 25(OH)D concentrations but have not consistently improved postoperative recovery [10,26,27]. The 2024 Endocrine Society guideline recommends daily rather than intermittent high-dose supplementation when treatment is indicated but does not define outcome-specific target serum 25(OH)D concentrations [10]. After hip fracture, supplementation should therefore be individualized according to vitamin D status, calcium intake, renal function, nutritional status, and planned osteoporosis therapy [10,24,25,27]. Calcifediol bypasses hepatic 25-hydroxylation and generally raises serum 25(OH)D faster and more predictably than cholecalciferol or ergocalciferol. This pharmacokinetic difference may be useful when rapid correction is desired, but the Cuadra-Llopart trial did not establish superior long-term functional outcomes for the faster daily regimen [22].

### 7.5. Integration into Secondary Fracture Prevention

Vitamin D supplementation should be considered one component of comprehensive secondary fracture prevention rather than an isolated intervention. Current recommendations emphasize post-fracture evaluation, fall-risk assessment, bone-density testing, osteoporosis treatment, and calcium/vitamin D supplementation when appropriate [32]. A quality-improvement initiative using standardized discharge orders increased the use of osteoporosis medication and calcium/vitamin D supplementation; the study did not evaluate whether this translated into improved long-term clinical outcomes [31].

## 8. Clinical Implications and Research Priorities

### 8.1. Clinical Interpretation of Vitamin D Status

Low serum 25(OH)D should be interpreted within the broader clinical context of malnutrition, reduced mobility, sarcopenia, renal dysfunction, cognitive impairment, comorbidity, and frailty rather than as an isolated laboratory abnormality [4,6,12,13,16,17,18,19,20]. Severe deficiency (approximately ≤10–12 ng/mL) appears to have greater prognostic relevance than mild insufficiency for selected functional outcomes, although heterogeneous thresholds, sampling methods, and outcome measures preclude the definition of a universal prognostic cutoff [17,18].

The 2024 Endocrine Society guideline does not recommend routine 25(OH)D screening in generally healthy adults without an established indication [10]. However, these recommendations should not be extrapolated directly to patients with recent osteoporotic hip fracture, who frequently have osteoporosis, hypocalcemia, secondary hyperparathyroidism, malnutrition, renal dysfunction, or other conditions affecting bone metabolism.

Accordingly, targeted assessment of serum 25(OH)D is most appropriate when the results may influence clinical management. Interpretation should consider calcium, albumin, parathyroid hormone, renal function, nutritional status, pre-fracture mobility, cognition, muscle status, and overall frailty [10,12,31].

### 8.2. Integration into Multidisciplinary Post-Fracture Care

Vitamin D correction should be incorporated into comprehensive post-fracture care rather than used as a stand-alone intervention. Current evidence supports combining vitamin D supplementation with rehabilitation, adequate protein and calcium intake, osteoporosis treatment, fall prevention, and management of sarcopenia rather than relying on dose escalation alone [27,28,29,30,31,33].

Consensus recommendations advise daily vitamin D supplementation (≥800 IU) for older adults with hip fracture, together with calcium supplementation when dietary intake is insufficient [33]. Treatment should be individualized according to vitamin D status, renal function, calcium balance, nutritional status, malabsorption, secondary hyperparathyroidism, and planned osteoporosis therapy [10,27,28,33].

System-level approaches, including fracture liaison services, standardized discharge pathways, multidisciplinary collaboration, and longitudinal follow-up, are essential to improve treatment initiation and long-term adherence [31,32,33].

### 8.3. Research Priorities

Future studies should adopt standardized definitions of vitamin D deficiency, distinguish severe deficiency from mild insufficiency, and standardize the timing and methodology of serum 25(OH)D measurement [2,3,4,8,9,10,11,12,13,16,17,18,19,20,21]. Observational studies should use standardized patient-centered outcomes, directly measure frailty and sarcopenia, and employ prespecified causal models to better distinguish confounding from biological mediation [2,3,4,6,9,12,13,16,17,18,19,20].

Randomized trials should preferentially enroll patients with confirmed, particularly severe, vitamin D deficiency and evaluate vitamin D supplementation within contemporary multidisciplinary hip fracture care. Future studies should compare standard replacement with alternative dosing strategies while accounting for calcium intake, rehabilitation, protein supplementation, osteoporosis therapy, and treatment adherence [17,18,23,27,28,30].

Finally, adequately powered multicenter trials should determine whether targeted correction of severe vitamin D deficiency improves patient-centered outcomes—including functional recovery, falls, secondary fracture, institutionalization, quality of life, and mortality—and whether integrated post-fracture care pathways further enhance these benefits [17,18,23,24,25,26,27,28,30,31,32,33].

## 9. Conclusions

Vitamin D deficiency is highly prevalent among older adults with hip fracture, but its clinical significance varies according to the severity of deficiency and the outcome evaluated. Low perioperative serum 25-hydroxyvitamin D [25(OH)D] has been associated with selected early medical complications, mortality, impaired functional recovery, and secondary hip fracture; however, these associations are heterogeneous and less consistent for mild insufficiency than for severe deficiency (approximately ≤10–12 ng/mL). Differences in vitamin D thresholds, sampling methods, outcome definitions, patient populations, follow-up duration, and adjustment for frailty contribute substantially to this heterogeneity [2,3,4,8,9,13,16,17,18,19,20,21].

Accordingly, low serum 25(OH)D should be regarded as both a potentially modifiable metabolic abnormality and a marker of a broader bone–muscle–frailty phenotype rather than as a uniform independent cause of adverse outcomes. Interpretation of vitamin D status should therefore consider the overall clinical context, including calcium–parathyroid hormone homeostasis, nutritional status, renal function, pre-fracture mobility, cognition, and frailty [5,6,7,11,12].

Although vitamin D supplementation consistently improves biochemical vitamin D status, current evidence does not demonstrate that supplementation alone reliably improves postoperative mobility, reduces complications or mortality, or prevents secondary hip fracture. Vitamin D assessment and correction should therefore be integrated into comprehensive post-fracture care that includes adequate calcium and protein intake, rehabilitation, fall prevention, osteoporosis pharmacotherapy, and longitudinal follow-up [23,24,25,26,27,28,29,30,31,32,33].

Future prospective studies and randomized trials should focus on patients with confirmed severe vitamin D deficiency, use standardized definitions and sampling protocols, and evaluate patient-centered outcomes with rigorous adjustment for frailty and rehabilitation exposure. The key unanswered question is not whether low vitamin D accompanies poor recovery, but whether targeted correction of severe deficiency within an integrated multidisciplinary post-fracture pathway can meaningfully improve functional recovery, complications, secondary fracture, and survival.

## Data Availability

No new datasets were generated in this study. The data used to illustrate the national trends in hip fracture burden are publicly available from the Health Insurance Review & Assessment Service (HIRA) Healthcare Bigdata Hub (https://opendata.hira.or.kr/). All other data discussed in this narrative review are available in the cited published literature.

## Supplementary Materials

The following supporting information can be downloaded at: https://www.mdpi.com/article/10.3390/medicina62091794/s1, Table S1: Targeted supplementary PubMed search strategies conducted on 18 July 2026, without publication-date restrictions.
