# Supplementary material for: Vitamin D Deficiency in Hip Fracture Patients: Outcome-Specific Associations and Post-Fracture Management—A Narrative Review
Source: Medicina (Kaunas). 2026 Sep 17;62(9):1794. doi: 10.3390/medicina62091794 (PMC13609279; doi:10.3390/medicina62091794)
Supplement: Supplementary file 1 [file medicina-62-01794-s001.zip › medicina-4539771-supplementary.pdf]

SUPPLEMENTARY MATERIAL

Supplementary Table S1. Targeted supplementary PubMed search strategies conducted on July 18, 2026, without publication-date restrictions.

Search ID	Purpose and exact PubMed strategy	Records
**S01-1**	**Purpose:** To identify prospective evidence on functional recovery and mobility after hip fracture in relation to vitamin D status.<br>**Exact PubMed strategy:**<br>(<br>"hip fracture"[Title]<br>OR "hip fractures"[Title]<br>)<br>AND<br>(<br>"vitamin D"[Title/Abstract]<br>OR "vitamin D deficiency"[Title/Abstract]<br>OR "25-hydroxyvitamin D"[Title/Abstract]<br>OR "25(OH)D"[Title/Abstract]<br>)<br>AND<br>(<br>"functional recovery"[Title/Abstract]<br>OR "functional outcome"[Title/Abstract]<br>OR "functional outcomes"[Title/Abstract]<br>OR mobility[Title/Abstract]<br>OR ambulation[Title/Abstract]<br>OR "walking ability"[Title/Abstract]<br>OR rehabilitation[Title/Abstract]<br>OR "quality of life"[Title/Abstract]<br>)	112
**S02**	**Purpose:** To identify evidence on postoperative complications and mortality, including null or inconsistent findings, in relation to vitamin D status after hip fracture.<br>**Exact PubMed strategy:**<br>(<br>"hip fracture"[Title]<br>OR "hip fractures"[Title]<br>)<br>AND<br>(<br>"vitamin D"[Title/Abstract]<br>OR "vitamin D deficiency"[Title/Abstract]<br>OR "25-hydroxyvitamin D"[Title/Abstract]<br>OR "25(OH)D"[Title/Abstract]<br>)<br>AND<br>(<br>mortality[Title/Abstract]<br>OR death[Title/Abstract]<br>OR survival[Title/Abstract]<br>OR prognosis[Title/Abstract]<br>OR "postoperative complication"[Title/Abstract]<br>OR "postoperative complications"[Title/Abstract]<br>OR "postoperative outcome"[Title/Abstract]<br>OR "postoperative outcomes"[Title/Abstract]<br>OR "clinical outcome"[Title/Abstract]<br>OR "clinical outcomes"[Title/Abstract]<br>OR delirium[Title/Abstract]<br>OR pneumonia[Title/Abstract]<br>OR infection[Title/Abstract]<br>OR "length of stay"[Title/Abstract]<br>)	158
**S03**	**Purpose:** To identify intervention studies of post-fracture vitamin D supplementation or dosing in patients undergoing hip fracture surgery.<br>**Exact PubMed strategy:**<br>(<br>"hip fracture"[Title]<br>OR "hip fractures"[Title]<br>)<br>AND<br>(<br>"vitamin D"[Title/Abstract]	93

Search ID	Purpose and exact PubMed strategy	Records
	OR cholecalciferol[Title/Abstract]<br>OR calcifediol[Title/Abstract]<br>OR ergocalciferol[Title/Abstract]<br>)<br>AND<br>(<br>supplementation[Title/Abstract]<br>OR supplemented[Title/Abstract]<br>OR "vitamin D treatment"[Title/Abstract]<br>OR "vitamin D therapy"[Title/Abstract]<br>OR "vitamin D administration"[Title/Abstract]<br>OR "vitamin D dose"[Title/Abstract]<br>OR "loading dose"[Title/Abstract]<br>)<br>AND<br>(<br>mortality[Title/Abstract]<br>OR survival[Title/Abstract]<br>OR function[Title/Abstract]<br>OR functional[Title/Abstract]<br>OR mobility[Title/Abstract]<br>OR walking[Title/Abstract]<br>OR rehabilitation[Title/Abstract]<br>OR complication[Title/Abstract]<br>OR complications[Title/Abstract]<br>OR outcome[Title/Abstract]<br>OR outcomes[Title/Abstract]<br>OR readmission[Title/Abstract]<br>OR "length of stay"[Title/Abstract]<br>OR falls[Title/Abstract]<br>)	
**S04**	**Purpose:** To identify evidence on vitamin D cutoff heterogeneity and mortality after hip fracture.<br>**Exact PubMed strategy:**<br>(<br>"hip fracture"[Title]<br>OR "hip fractures"[Title]<br>)<br>AND<br>(<br>"vitamin D"[Title/Abstract]<br>OR "25-hydroxyvitamin D"[Title/Abstract]<br>OR "25(OH)D"[Title/Abstract]<br>)<br>AND<br>(<br>cutoff[Title/Abstract]<br>OR "cut-off"[Title/Abstract]<br>OR threshold[Title/Abstract]<br>OR deficiency[Title/Abstract]<br>OR insufficiency[Title/Abstract]<br>OR "severe deficiency"[Title/Abstract]<br>)<br>AND<br>(<br>mortality[Title/Abstract]<br>OR survival[Title/Abstract]<br>OR prognosis[Title/Abstract]<br>OR outcome[Title/Abstract]<br>OR outcomes[Title/Abstract]<br>)	49
